# Faber’s Talking Machine

**Published:** 1872-07

**Authors:** Ralph M. Townsend


*Faber's Talking Machine.* By RALPH M. TOWNSEND, M.D.

A machine that utters the articulate sounds of the human voice has lately been exhibited in this city, the initial exhibitions being given at the Jefferson Medical College and the University of Pennsylvania respectively. On both of these occasions an interesting accompanying lecture on "The Organs of Voice and the Mechanism of Speech," was delivered by J. Solis Cohen, M.D.

Joseph Faber (uncle of the present exhibitor of the apparatus), a former Professor of Mathematics in Vienna, constructed this machine as an exact copy, so far as was possible, of the human vocal apparatus. It was exhibited here thirty years ago, since which time the present Prof. Faber has remodeled and improved it. Its mechanism is intricate, resembling, in its labyrinth of springs, strings, wires, and tubing, a loom, and in the complexity of its workings, the Alden type-setter.

The machine proper is mounted upon a table and operated by a key-board and levers, like a piano. Under the table is a treadle that works the bellows, supplying air to the larynx. The latter is formed of india-rubber, with a movable glottis of thin lamellæ of ivory to give the necessary tension. The upper jaw is wooden and stationary, having a lip of leather. The under jaw, which is movable, is made of gutta-percha, as is the roof of the mouth. The tongue is made of rubber, is flexible and movable, and can be pressed either against the palate or back into the throat. As the mouth is unprovided with teeth, a narrow metallic band is made to fall from over the upper lip and close the fissure of the mouth whenever the dental sounds are required. There is no soft palate. The nasal opening which permits the production of the sounds of *m* and *n* is formed by a tube proceeding from the larynx, beyond the vocal cords, and when the lips are closed, as for *b*. In the same way the *n* is made by the *d*. A little shuttle-wheel is so contrived as to be dropped into the current of air proceeding to the glottis, where its rapid revolution produces the sound *r*.

The lungs are represented by a pair of bellows. The instrument speaks phonetically, and therefore can utter words in any language. Fourteen sounds are used—viz., the vowels, *a*, *e*, *i*, *o*, *u*, and the consonants, *l*, *r*, *w*, *f*, *s*, *sh*, *b*, *d*, and *g*. In some instances remaining sounds are obtained by variously combining these, and in others they are modified by opening the glottis. The operator, Madame Faber, being a German and not speaking a word of English, the accent of the former tongue is readily distinguishable in the utterance of the machine. A mask, with a tubular nose, is slipped over the mouth when the machine speaks French, so as to give the nasal sound peculiar to the pronunciation of that language. The instrument is also able to speak in a very high or in a deeper tone; but all parts of a complete sentence must be intonated alike.

Such, in brief, are the appearance and construction of this mechanical marvel. Its utterances are distinct, although monotonous and sepulchral—totally devoid of modulation, emphasis, or shade of expression. Its emotional impressions rank with those of Mrs. Shelly's "Frankenstein"—a literary monster that has haunted the pillows of two generations of nocturnal readers. It affects a timid individual like a gibbet and skeleton creaking and rattling in the night wind; and one does not marvel at the practical deductions of a Huxley or a Darwin after hearing it drawl out, "I—can—talk—as—well—as—anybothy—buth—I'm—a—ma—sheen." *

As a fruit, however, of man's patience and ingenuity—as an apparatus illustrative of the workings of the vocal organs—and, finally, as showing the infinite superiority of God's handiwork over man's most elaborate imitations—in all of these this machine stands forth in pleasantly instructive lights, and therefore has its lessons that cannot be too well studied.

In his lecture, Dr. Cohen gave a short sketch of the history of these talking machines, an abstract of which will not prove uninteresting:

"Although attempts at the artificial production of speech had been long made, the first partial success seems to have dated from 1779, in which year the Imperial Academy of Sciences at St. Petersburg proposed, as the subject of one of their annual prizes, an inquiry into the nature of the (Continental) vowel-sounds—*a*, *e*, *i*, *o*, and *u*—with the construction of an instrument imitating them artificially. This prize was awarded to one Kratzenstein, who showed that the vowel-sounds could be distinctly produced by blowing through a reed in the lower ends of pipes of certain formation—one for each vowel.

"About the same time, Wolfgang von Kempelen, of Automaton Chess-Player notoriety, attempted the construction of a talking machine, in which he was moderately successful. The vowel-sounds were produced by adapting a reed to the lower portion of a funnel-shaped tube, the sounds of which were modified by inserting the fist into the mouth of the funnel. He afterwards constructed an oval-shaped box which opened on hinges so as to represent the action of a jaw. The tube containing the reed entered this box, and by opening and closing the jaws he produced the sounds *a*, *o*, *u*, in a satisfactory manner; *e* was imperfect, and he was unable to produce the sound *i*. (These are the Continental vowel-sounds.)

"Subsequently, he was enabled to obtain from different jaws the sounds of the consonants *p*, *m*, *l*, and, by means of combining these, he was enabled to deceive the ear by an imitation of most of the other consonant sounds; and, finally, he completed an arrangement with only one mouth and one glottis, the mouth consisting of a funnel-shaped piece of india-rubber, the sides of

* In support of Darwin's theories the machine might add, but Dr. Darwin—theths—I may—if I behave myselthf—become—a man.—Ed.

which were compressed by the fingers, so as to represent the lips in the formation of *b*, *p*, *m*, and *v*. In the tube leading from the wind-chest into this mouth were two tin tubes, capable of being opened by compressing a button at their free extremity, and thus representing the nostrils. When both of these were open, and the mouth closed, he produced the sound *m*, and when only one of them was open, he produced an *n*. Three valves were upon the wind-chest, two of which when open produced the sounds *s* and *sh*, respectively, made by little pipes; and the third valve when opened permitted the vibration of a separate reed which made a sound somewhat resembling the roll of the *r*. The whole apparatus was enclosed in a box about three feet in length and rectangular in form. It was placed upon a table, and covered with a cloth, under which the inventor placed his hands while operating the machine. He was able to produce a great number of words and sentences—among others, *papa*, *mamma*, *lama*, *aura*, *opera*, *astronomy*, *Constantinople*, *exploitation*, *vous etes mon ami*, *je vous aime de tout mon cœur*, *venez avec moi a Paris*, *Leopoldus secundus*, *Romanorum imperator semper Augustus*, etc.

"Mr. Willis, of Cambridge, repeated the experiments of Kempelen, using a shallower funnel, and, instead of introducing his hand to modify the sounds, succeeded in producing the whole series of vowel-sounds by sliding a flat board more or less over the top of the funnel. He then adapted tubes to the reed, and varied the length of the tube by telescopic slides. When the tube was shorter than that of a stopped pipe in unison with the reed, he produced an *i*, and by increasing the length of the tube the sound changed in succession to *e*, *a*, *o*, and *u*. When the tube was lengthened so far as to be half as long again as the length of a stopped pipe, in unison with the reed, the vowels again were sounded, but in a reversed order, and then again in direct order when the length of the tube was equal to twice that of a stopped pipe in unison with the reed."

After this came the original invention of Professor Faber, which, with its later modifications, I have above described.—*Medical Times.*

---

### *Impotency.*

Prof. D. Hayes Agnew, of Philadelphia, (*Medical and Surgical Reporter*), in a clinical lecture on impotency, after stating that the treatment must be largely moral, and urging the abandonment of onanism or excessive venery, says that there is no better internal treatment than phosphorus and strychnia—1-50 grain of the former, and 1-30 of the latter—made into a pill, and administered three times daily.—*Clinic.*

